# Towards Precision Vaccines: Lessons From the Second International Precision Vaccines Conference

**DOI:** 10.3389/fimmu.2020.590373

**Published:** 2020-10-15

**Authors:** Dheeraj Soni, Simon D. Van Haren, Olubukola T. Idoko, Jay T. Evans, Joann Diray-Arce, David J. Dowling, Ofer Levy

**Affiliations:** ^1^Precision Vaccines Program, Division of Infectious Diseases, Boston Children’s Hospital, Boston, MA, United States; ^2^Department of Pediatrics, Harvard Medical School, Boston, MA, United States; ^3^Vaccine Centre, London School of Hygiene and Tropical Medicine, London, United Kingdom; ^4^Center for Translational Medicine, University of Montana, Missoula, MT, United States; ^5^Broad Institute of MIT & Harvard, Cambridge, MA, United States

**Keywords:** precision vaccines, systems biology, International Precision Vaccines Conference, vaccinologists, non-infectious diseases, adjuvants, formulations, vulnerable populations

## Abstract

Other than clean drinking water, vaccines have been the most effective public health intervention in human history, yet their full potential is still untapped. To date, vaccine development has been largely limited to empirical approaches focused on infectious diseases and has targeted entire populations, potentially disregarding distinct immunity in vulnerable populations such as infants, elders, and the immunocompromised. Over the past few decades innovations in genetic engineering, adjuvant discovery, formulation science, and systems biology have fueled rapid advances in vaccine research poised to consider demographic factors (*e.g.*, age, sex, genetics, and epigenetics) in vaccine discovery and development. Current efforts are focused on leveraging novel approaches to vaccine discovery and development to optimize vaccinal antigen and, as needed, adjuvant systems to enhance vaccine immunogenicity while maintaining safety. These approaches are ushering in an era of precision vaccinology aimed at tailoring immunization for vulnerable populations with distinct immunity. To foster collaboration among leading vaccinologists, government, policy makers, industry partners, and funders from around the world, the *Precision Vaccines Program* at Boston Children’s Hospital hosted the 2^nd^ International Precision Vaccines Conference (IPVC) at Harvard Medical School on the 17^th^–18^th^ October 2019. The conference convened experts in vaccinology, including vaccine formulation and adjuvantation, immunology, cell signaling, systems biology, biostatistics, bioinformatics, as well as vaccines for non-infectious indications such as cancer and opioid use disorder. Herein we review highlights from the 2^nd^ IPVC and discuss key concepts in the field of precision vaccines.

## Introduction

Vaccines remain the most effective public health intervention to reduce the burden of infectious diseases. However, several factors can influence and ultimately alter the efficacy of vaccines. These include differences in immune status (*e.g.* healthy *vs* immunocompromised individuals), sex, and age (*e.g.* newborn/infant *vs* adult *vs* elderly) (1). The significance of this disparity is illustrated by the relatively high burden of infections at the young and the elderly compared with middle-aged adults. Furthermore, newborn and young infants (<6 months of age) display distinctions in immune cell functionality that creates a “window of vulnerability”, making some vaccines less effective in this group as compared to the same strategies used later in life (2, 3). In addition, there are numerous other factors which may influence immune responses and consequently affect vaccine efficacy. These can be circadian and circannual rhythms as well as geographical location which can correspond to individuals with distinct genetic and epigenetic backgrounds (4). It is imperative to consider this multitude of factors for developing vaccines that target pathogens endemic to specific geographical area. Finally, in considering vaccine (self-) adjuvantation, whole, live microorganisms activate distinct immune responses that are typically more robust than those induced by individual adjuvants (5, 6). The use of optimized adjuvanted vaccine formulations targeted to a given population may overcome barriers in vaccine development and match or even exceed pathogens in eliciting effective immune responses (7).

**Figure 1:**
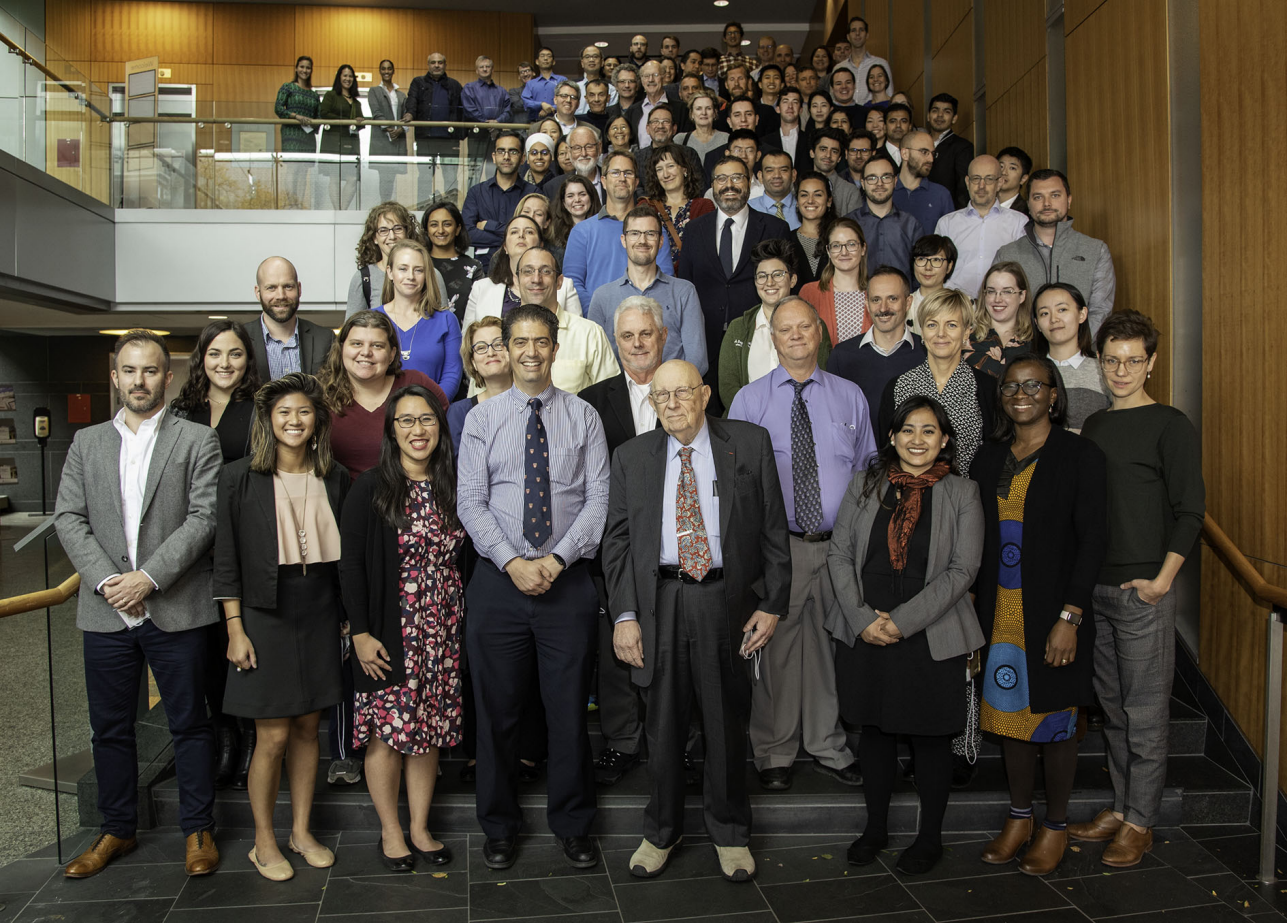
Attendees of the 2^nd^ biennial International Precision Vaccines Conference. The 2^nd^ biennial IPVC (17^th^ and 18^th^ October 2019), sponsored by the Boston Children’s Hospital *Precision Vaccines Program*, was held at the Joseph B. Martin Conference Center at Harvard Medical School (Boston, MA, USA).

To discuss strategies and foster synergies in vaccine development, a community of experts from a range of fields, including immunology, pediatrics, vaccinology, systems biology utilizing powerful big data (“OMIC”) approaches, as well as vaccine adjuvantation and formulation, gathered at the Joseph B. Martin Conference Center at Harvard Medical School (Boston, MA, USA) on the 17^th^ and 18^th^ October 2019 for the 2^nd^ biennial International Precision Vaccines Conference (IPVC) (**Figure 1**). The following sections report highlights from the conference followed by key concepts in the field of precision vaccines.

### Precision Vaccines Program

After brief welcoming remarks from Dr. Gary R. Fleisher (Department of Medicine, Boston Children’s Hospital and Harvard Medical School, Boston, MA, USA), Dr. Ofer Levy (*Precision Vaccines Program*, Boston Children’s Hospital and Harvard Medical School, Boston, MA, USA) introduced the *Precision Vaccines Program* (PVP) that sponsored the conference (http://www.childrenshospital.org/research-and-innovation/research/departments/medicine/precision-vaccines-program). Based in the Division of Infectious Diseases at Boston Children’s Hospital, the PVP fosters international collaboration to characterize distinct vaccine-induced immune responses of vulnerable populations such as the very young and the elderly to inform development of novel vaccines tailored to protect them. Program members have domain expertise in vaccinology, clinical trials, immunology, molecular biology, biostatistics, bioinformatics, and powerful big data (“OMIC”) approaches.

Recent advances in genetics, molecular and systems biology, as well as in translational medicine have informed a precision medicine strategy for defining subpopulations of patients sharing similar characteristics and tailoring medical interventions according to a patient’s responsiveness. The use of this approach in vaccinology, further enhanced by advances in immune ontogeny studies and human *in vitro* culture systems, as well as in adjuvantation and formulation science, is paving the way for the development of precision vaccines. These are defined as vaccines that (i) consider the target population; (ii) are formulated to selectively activate the immune system by targeting specific anatomic sites, cells, and pathways that generate a protective response; and (iii) may, as needed, contain adjuvantation systems known to optimally enhance immunogenicity in a target population. In order to accomplish this complex and crucial task, systems biology methodologies such as transcriptomics, proteomics, and metabolomics as well as preclinical human *in vitro* models that consider age- and sex-specific differences can be leveraged to generate hypotheses to be tested in appropriate animal models and eventually in targeted clinical trials. Key to the success of this approach is interdisciplinary collaboration such as that catalyzed by the PVP and IPVC.

Since the 1^st^ IPVC held in 2017 (8), PVP has experienced a marked growth, including (a) a robust Precision Vaccines Network now >400 individuals from academia, government, and industry, (b) growth in the scope of our collaborative systems biology and immune ontogeny studies on vaccines against hepatitis B, influenza, HIV, RSV and pertussis as well as opioid overdose, and (c) a growing stream of innovation in discovery and development of adjuvants and adjuvanted vaccines.

This 2^nd^ Biennial Precision Vaccines Conference served as a platform to foster international collaboration for developing vaccines tailored to distinct and vulnerable populations such as the young and elderly. The target audience included academic-, government-, and industry-based physicians, scientists, and trainees interested in developing vaccines for vulnerable populations.

### Horizon for New Vaccine Development

Dr. Stanley A. Plotkin (University of Pennsylvania, Philadelphia, USA) opened the conference by emphasizing the need for precision vaccinology. Dr. Plotkin highlighted widely used approaches for developing attenuated (*i.e.* physical changes or passage in animals/eggs/cell culture) and inactivated (killed whole organisms and utilizing polysaccharides or purified proteins) vaccines (9). However, growing knowledge in the field of vaccinology has established that host genetics also plays a vital role in vaccine responses, therefore, to tackle new complex problems we need to utilize innovative strategies. Attenuated vaccines include temperature-sensitive mutations and reassortment, viral recombinants and deletion mutants, codon de-optimization, microRNA insertion, and replication vectors that present genes from pathogens. Novel strategies for developing inactivated vaccines include DNA plasmids, mRNA, reverse vaccinology, antigen identification by transcriptomics and proteomics, structural analysis, adjuvants, and induction of innate immunity. Dr. Plotkin highlighted the importance of systems biology approach for a rational vaccine design (10). In addition, he identified currently unsolved problems in vaccinology including (a) immune memory; (b) multiplicity of virulence antigens in complex pathogens; (c) multiple HLA types; (d) conserved epitopes; (e) finding correlates of protection; (f) immaturity and post-maturity of the immune system; (g) mucosal immunization with non-replicating antigens; (h) adjuvants capable of selectively expanding cell types; and (i) the challenge of generating T-cell immunity without replicating vaccines. The possible solutions include (a) enhancing stimulation Tfh cells and induction of innate immunity by TLR agonists; (b) analysis of natural immune responses *i.e.*, “antigenomics”; (c) developing polyepitope vaccines; (d) utilizing structural biology; (e) systems biological approaches; (f) cytokine modulation; (g) formulation approaches, specifically nanoemulsions; (h) using single or combined TLR ligands; and (i) utilizing toolbox of adjuvants; respectively (11). Many of these problems along with their potential solutions were discussed in detail during the conference.

Dr. Dan Barouch (Center for Virology and Vaccine Research at Beth Israel Deaconess Medical Center; Harvard Medical School) provided an overview of the recent progress in preclinical to clinical development of HIV vaccines. Although significant progress has been made with dozens of highly effective anti-HIV drugs available to control the infection, there is an unmet need for an HIV vaccine to effectively address the growing HIV/AIDS pandemic (12). Dr. Barouch provided an update on currently ongoing efficacy trials for two HIV-1 vaccine candidates and a broadly neutralizing mAb. The strategy for a global prophylactic HIV-1 vaccine includes the use of: a) vectors that elicit potent immune responses in individuals irrespective of their spatial location, b) bioinformatically engineered antigens (a.k.a mosaic antigens) to improve immunologic coverage of global virus diversity, and c) envelope proteins to increase humoral immunity. These trials are based on the foundation of precision vaccines, with the ultimate aim of identifying immune responses which would aid in protection against the infection (13). He presented work on mosaic HIV-1 vaccines (*i.e.* Ad26/Env) which provided up to 40–67% protection against SIVmac251 and SHIV-SF162P3 challenges in non-human primates (NHPs). In addition, adoptive transfer of purified IgG from Ad26/Env vaccinated NHPs provided protective efficacy against SIVmac251 challenges (13, 14). Since these data suggest that functional antiviral antibodies are responsible for protection against SIVmac251 in NHPs, current ongoing studies involving phase-2b/3 clinical efficacy trials are aimed at evaluating the efficacy of functional antiviral antibodies for protection in humans against HIV-1.

#### Vaccines for Vulnerable Populations: Ontogeny and Immunodeficiency

Dr. Richard Malley (Boston Children’s Hospital; Harvard Medical School; Affinivax) provided an overview of the novel vaccine platform named multiple antigen presenting system (MAPS) and highlighted an example of it's application to develop vaccines against *Staphylococcus aureus*. His earlier research focused on developing broad serotype-independent protection against pneumococcal disease identified three key challenges: a) serotype replacement, b) cost (complex and difficult to manufacture vaccine), and c) immunogenicity does not always imply clinical efficacy (15). Although killed whole cell vaccine (WCV) is cheaper to manufacture and addresses serotype replacement, it comes with distinct challenges such as a) growing hesitancy for WCVs in vulnerable populations (*i.e.* infants), b) inapplicability to a variety of pathogens, and c) high immunogenicity due to particulate nature of WCVs. Therefore, Dr. Malley et al. have developed MAPS which induces broader responses (both B-cell and T-cell) with several potential immunological, technological, and financial advantages (16). MAPS enables the creation of a macromolecular complex that mimics the properties of WCVs by integrating various antigen components (including proteins and polysaccharides) within the same construct to aid in inducing multipronged immune responses including, Th1 and Th17 responses (16).

Dr. Paolo Palma (Bambino Gesù Children’s Hospital, Rome, Italy) highlighted how vaccination coverage is lower in vulnerable populations (VPs) and/or individuals with chronic conditions, particularly due to lack of safety and immunogenicity data in the specific populations. However, research in the past decade has led to the switching of vaccine development from empirical to a personalized vaccinology approach, which promises to advance effective treatments for VPs. Dr. Palma’s laboratory has made several observations emphasizing that correlates of protection in individuals with transplants (17) or chronic conditions (such as HIV-infection) are significantly different from the healthy individuals (18–20). In conclusion, he highlighted the virtuous circle of precision vaccinology which involves data collection from existing cohorts, data modeling of VPs, development of a harmonized OMICs platform, vaccine trials in VPs and identification of novel biomarkers of immunogenicity and safety to tailor vaccine intervention to those who may most benefit from its considerable promise (21).

#### Trained/Heterologous Immunity

Dr. Christine Benn (Statens Serum Institut; Denmark) discussed research activities from the Bandim Health Project in Guinea-Bissau and the Danish Institute for Advanced Studies, University of Southern Denmark (22). This included the non-specific (heterologous) effects of vaccines in Africa and Europe (23). She discussed how non-specific effects of vaccines on other diseases may impact overall health and the differences between these effects apparently related to the differential effects of live (*e.g.*, BCG) *vs* non-live [*e.g.*, diphtheria–tetanus–pertussis (DTP)] vaccines. Her studies have suggested that live vaccines tend to induce beneficial overall non-specific effects, whereas non-live vaccines tend to induce negative non-specific effects, especially in girls. She emphasized the urgent need for a better understanding of the mechanisms underlying non-specific vaccine effects (24). She highlighted that current vaccines are largely understood within the framework of their specific effects and that a real-life assessment of overall effects of vaccines is urgently needed. She viewed the following as key to developing new vaccines: a) test vaccines *vs* true placebo and studying overall health effects pre-licensure b) randomized implementation, c) developing methods for post-licensure evaluation, d) developing live vaccines or artificially mimicking effect of live vaccines; and e) changing focus from preventing single diseases towards enhancing overall host health.

Dr. David J. Dowling (PVP, Boston Children’s Hospital; Harvard Medical School) presented an overview of ongoing work within the PVP to exploit the concepts of trained/heterologous immunity into next generation adjuvanted vaccine formulations (25, 26). Adjuvantation is a key approach to enhancing vaccine immunogenicity (27). For example, Dr. Dowling highlighted the need to characterize the mechanisms underlying heterologous immunological responses elicited by the BCG vaccine (28) to inform rational design of synthetic formulations that mimic BCG’s heterologous beneficial effects while avoiding and improving upon its shortcomings (29). Both the immunological pathways and duration of immunostimulation elicited by BCG vaccination have been linked to its efficacy as a neonatal vaccine. As such, study of BCG-induced innate memory may inform discovery and development of novel vaccine adjuvants. In addition, he reviewed prior work from the PVP in which a) BCG was shown to act as an adjuvant, in an age- and formulation-specific fashion (30), b) BCG formulations are not uniform (31) and c) TLR8 may mediate BCG vaccine-induced protection, and TLR8 agonist-containing nanoparticle delivery systems designed to mimic BCG efficiently induce trained immunity in newborn mice (32).

#### Clinical Aspects

This session included discussions on clinical considerations in vaccinology and highlighted research activities from the speakers from their research studies across the globe.

Dr. Lindsey Baden (Brigham and Women's Hospital; Dana Farber Cancer Institute; Harvard Medical School; Ragon Institute of MGH, MIT and Harvard) highlighted the challenges in vaccine development for various vaccines currently in trials. These challenges include inadequate knowledge of correlates of protection for novel vaccines, the need to optimize a range of factors for optimal immunogenicity, including dose, schedule, route, delivery system, and adjuvants to elicit potent immune responses, as well as global diversity and vaccine manufacturing. He highlighted how several HIV vaccine trials to date have failed to demonstrate beneficial efficacy (33, 34). In addition, he discussed the development of Mosaic Ad26/Env HIV-1 vaccine which is a multi-year study in collaboration with Dr. Dan Barouch and is currently advanced to Phase 3 trial (13). Dr. Baden also shared data from multiple studies including CMV (35) and cholera (36) vaccines, to highlight the need for leveraging a proper model for vaccine developing since: a) different pathogens pose very different challenges, b) both science and product development can occur simultaneously, and c) developing proper models or surrogates with clinical meaning facilitates rapid iteration along with informing the meaning of immunologic parameters measured.

Dr. Nadine Rouphael (Emory University, Atlanta, USA) highlighted the research conducted at Hope Clinic Mission of Emory University aimed at translating basic research discoveries to clinical advances. This included research targeted at unravelling the differences in vaccine responses based on age, environmental conditions (*e.g.*, role of stress, diet, infection, microbiome) and genetic differences (37). In addition, she highlighted the potential of systems vaccinology in probing diversity among human immune systems, by delineating the impact of the genes, the environment, and the microbiome on vaccination-induced protective immunity. Such insights are crucial, for example, for optimizing vaccines for immune-compromised populations (38).

Dr. Olubukola Idoko (PVP; Medical Research Council, The Gambia) highlighted research from the Medical Research Council Unit at the London School of Hygiene and Tropical Medicine in The Gambia (West Africa), including the long-standing contributions to pneumococcal vaccine and epidemiology research, spanning 40 years. She highlighted some challenges with conducting trials in the developing world including the challenges of obtaining informed consent on account of linguistic diversity and the limited clinical and trial facilities, while stressing the need for trials in these settings particularly as evidence indicates that vaccines work differently in different settings. She presented an example of markedly varied responses to yellow fever vaccine in two West African settings and the absence of any demonstrable antibodies to yellow fever 5–6 years post vaccination in 20% of a cohort of African infants (39).

### Approaches to Enhance Vaccinology

#### Systems Biology

For the past decades, systems biology approaches have increased our understanding of molecular interactions in biological systems (40). High throughput technologies such as different ‘omics’ modalities have enabled interrogation of vital biological processes within the immune system and holistic prediction of system behaviors (41, 42). Systems vaccinology alludes to the application of systems biology to study vaccine discovery, development, and immunogenicity. Immune signatures measured prior to and after vaccination enable predicting modulated targets and inform optimized vaccination strategies (43). Systems biology experts at the IPVC elucidated how this approach can provide insight into the mechanisms of vaccine immunogenicity.

Dr John Tsang (NIH Center of Human Immunology and Multiscale Systems Biology Section NIAID, NIH, Bethesda, USA) presented the relationship of pre-*vs* post-vaccination neutralizing antibody titers as a variable characteristic in human immune responses (44, 45). He explained that while genetics could partially explain phenotypic variability in early life, additional non-genetic factors contribute to the complex state of individual human immunity. Machine learning techniques can be applied to build predictive models and uncover biologically relevant parameters such as the quality and quantity of an immune response following an intervention such as immunization. Dr. Tsang reiterated that one distinct advantage of the human model is having substantial population variations to power correlation analyses (46). His work on global analyses of human immune variation found that baseline predicts antibody responses independent of age and pre-existing antibody titers using pre-perturbation cell populations (44). These findings suggest that baseline differences between individuals may be a key determinant of distinct individual responses to an intervention providing a resource for studying human immunity in health and disease.

Dr. Scott Tebbutt (University of British Columbia; PROOF Center, Vancouver, BC, Canada) introduced the concept of multivariate methods for single and integrative multi-omics supervised analyses. He outlined his team’s involvement with the Human Vaccine Project for Hepatitis B vaccination and Varicella Zoster vaccination study in adults using multiple systems biology techniques. Since the scale of these studies is enormous, combining datasets enables identification of systems biology features that are broadly predictive of antibody response. He found that combined datasets without scaling and batch correction resulted only in modest performance, while scaled/ComBat-corrected concatenated datasets were ten times improved with performance. The study revealed genes relating to T-cell regulated biology as the top enriched pathway. A single gene feature RAB11 Family interacting protein 5 (RAB11FIP5) was identified as important for induction of broadly neutralizing antibodies by the *Shingrix* zoster vaccine. Their work on multi-integration models may help identify predictors of antibody responses to multiple vaccines.

Dr. Al Ozonoff (PVP, Boston Children’s Hospital; Harvard Medical School) presented practical issues and solutions to digital infrastructure for systems biology. He outlined that responsibilities of the PVP-Data Management Core (PVP-DMC), which he leads, includes accurate and reliable data capture, secure project data management and analytic computing, and quality assurance. In addition to the importance of these approaches to achieving project's scientific goals, a successful computing infrastructure also enables a collaborative and integrative environment balancing centralized and de-centralized structures and processes. Data security is strictly ensured while striking a balance with data access to both computational savvy researchers and biologists without compromising data integrity. While cloud-based collaborative science is an attractive approach to collaborative systems biology studies, challenges and trade-offs should be discussed with collaborators early during project design.

Dr Madeleine Jennewein (Ragon Institute of MGH, MIT and Harvard) reported how systems serology is a powerful approach to dissect maternal antibody transfer to a newborn. Systems serology platform describes how biological samples lead to biophysical measurements of antigen-specific analyses as well as profiling the functional activities of numerous immune cells. Maternal vaccination protects neonates during the first year of life due to specialized antibody transfer across the placenta (47). The neonatal Fc receptor, FcRn, doesn’t fully account for antibody transfer. Indeed, the placenta selectively transfers NK cell-activating antibodies to neonates to access functional neonatal innate immune cells. Differential antibody Fc-glycosylation controls antibody transfer across the placenta and neonatal NK cells appear to be highly responsive to immune complexes. These findings may inform vaccination strategies to control antibody glycosylation to optimize the beneficial impact of maternal vaccination.

Dr. Robert Hancock (University of British Columbia, Vancouver, BC, Canada) illustrated how systems immunology approaches such as protein–protein interactions can infer functional links to understand inflammation, immunity, and sepsis. He presented the massive gene expression changes that occur in the first week of life in Gambian newborns. His team developed a web-based tool *NetworkAnalyst* to comprehensively allow researchers to determine features and functions leading to generation of biological hypotheses (48). With this tool, the same themes and pathways relating to interferon signaling, complement, and neutrophil activity are present in Gambian and Papua New Guinean newborns in the first week of life. They also applied this tool to a transcriptomics study of pediatric appendicitis, discovering a large number of significant differentially expressed genes between perforated and simple appendicitis involved in immunoregulatory interactions between a lymphoid and non-lymphoid cell, neutrophil degranulation as well as interleukin and interferon signaling. Dr. Hancock highlighted his group’s work on meta-analysis of sepsis biomarkers from 10 studies and found that early sepsis is associated with a diagnostic immune non-responsiveness gene expression signature related to immune cell reprogramming or endotoxin tolerance (49).

Dr. Jessica Lasky-Su (Channing Network Medicine, Brigham and Women’s Hospital; Harvard Medical School) presented how the plasma metabolome affects asthma pathophysiology. She presented the Maternal Vitamin D Supplementation to Prevent Childhood Asthma (VDAART) trial that is assessing whether vitamin D supplementation in pregnant women can prevent the development of asthma and allergies in offspring (50). Vitamin D plays a critical role in immune responses that may reduce inflammation in the airways and the likelihood of developing an infection (51). The VDAART study follows pregnant women from 10 to 18 weeks and their offspring through 6 years of age specifically assessing asthma related outcomes. Her group is also participating in the Copenhagen Prospective Studies on Asthma in Childhood (COPSAC) study that found that maternal vitamin D reduces the risk of asthma and wheezes. From these studies they have identified maternal metabolites associated with asthma in offspring at ages 3 and 4 years including the asthma bronchodilator theophylline. The presence of caffeine was associated with lower odds of asthma at 3 years of age. Her group found that high concentrations of polyunsaturated fatty acid (PUFA) and Vitamin D were associated with a large reduction in asthma risk (52). They also identified a potential role in asthma of the sphingolipid biosynthesis regulator ORMDL3. A combination of absence of a high risk ORMDL3 allele together with vitamin D supplementation was associated with the lowest risk of asthma suggesting that ORMDL3 is a key regulator of sphingolipid biosynthesis.

Dr. Hanno Steen (PVP, Boston Children’s Hospital; Harvard Medical School) reported on powerful sample sparing proteomic technologies that enable extraction of a deep proteome from sub-microliter volumes of plasma or serum. He outlined the objective to develop a plasma proteomics platform that is applicable to small samples, provides high throughput, and is cost efficient. He described a mass spectrometry-based workflow wherein as little as 0.3 µl of plasma or serum enables analysis to a robust analytical depth (53). He presented studies employing these methods to study small sample volumes in the context of immune ontogeny across the first week of life, Lyme Disease biomarker discovery as well as systems vaccinology to characterize responses to hepatitis B and influenza vaccines. This powerful and efficient approach is poised to make a positive impact across a wide range of studies in infectious diseases and vaccinology.

#### Vaccine Adjuvantation and Formulation

Vaccine adjuvants and formulation/delivery technologies have been a key driver in the advancement of modern vaccinology. For nearly 80 years aluminum salts were the only adjuvant included in FDA approved vaccines. This has changed in recent years with multiple vaccines containing new adjuvants approved including those with oil in water emulsions, such as MF59 and AS03 (54, 55), TLR agonists monophosphoryl lipid A and CpG (56, 57) and innovative adjuvant combinations including AS01 and AS04 (58, 59). Recent successes in the development and approval of new safe and effective adjuvants have changed the playing field and opened new doors for precision vaccines targeting vulnerable groups such as infants, elders, and immunocompromised populations. Experts at the 2^nd^ biennial IPVC highlighted a number of new adjuvant and formulation systems being developed in laboratories across the world.

Dr. Dennis Christensen (Statens Serum Institut, Denmark) presented on the role of depot formulations in the induction of systemic and mucosal immunity (60). Dr. Christensen and colleagues previously reported the utility of “prime-pul” vaccination (IM prime followed by intrapulmonary boost) in generating both systemic and mucosal responses to a TB subunit vaccine (H56) in combination with CAF01 adjuvant (trehalose-6,6-dibehenate (TDB) liposomes) (61, 62). The mechanisms for this enhanced immunity have been further elucidated by evaluating the uptake of H56/CAF01 by APCs and lung epithelial/endothelial cells after prime-pull vaccination or prime (IM or intrapulmonary) vaccination alone. Compared to single vaccinations by either route, prime-pul vaccination enhanced vaccine uptake by pulmonary endothelial and epithelial cells as well as pulmonary and splenic APCs leading to stronger activation of lung-draining lymph node DCs. Thus, innate and adaptive responses can be differently controlled through the priming and booster route of vaccination, timing of vaccinations, and use of adjuvants. These observations suggest that both innate myeloid APCs and immune memory play a role in prime-boost vaccine responses, and different immunization strategies can be employed to alter vaccine-induced immunity.

Dr. Christopher Fox (Infectious Disease Research Institute, Seattle, USA) highlighted the identification, characterization and testing of conifer-derived polyprenols as an alternative to squalene in oil-in-water nanoemulsions. Oil-in-water emulsions (AS01 and MF59) have been widely used to enhance immunity in the elderly and young children with >200 million doses administered to date. Squalene (derived from shark liver) is a major component in many oil-in-water nanoemulsions, including both AS01 and MF59 (54, 55), but very little is known about the structure–activity-relationship (SAR) for this important emulsifier. Dr. Fox reported that polyprenols derived from Siberian fir demonstrated favorable physiochemical properties in comparison to squalene and enhance immunity to an influenza virus vaccine in mice, ferrets, and pigs. Vaccine immunity was further enhanced in mice and ferrets with the addition of a synthetic TLR4-based adjuvant, GLA, to the polyprenol emulsion.

Dr. Simon van Haren (PVP; Boston Children's Hospital; Harvard Medical School) provided an update on the induction of neonatal Th1 and CD8 immunity to respiratory syncytial virus (RSV) using age-specific synergistic adjuvant combinations. Dr. Haren and the PVP team previously reported that newborn DCs are highly responsive to dual activation with trehalose-6,6-dibehenate (TDB), a Mincle receptor ligand and R848, and TLR7/8 ligand, driving a Th1 polarizing cytokine response (63). Dr. van Haren extended these published studies demonstrating that human cord blood derived DCs activated with R848 plus TDB synergistically activated signaling pathways that lead to antigen cross-presentation on MHC class I. The combination adjuvant (3M-052+TDB liposomes) was evaluated for adjuvant activity *in vivo* using the RSV pre-fusion F antigen. Administration of a vaccine including this dual-adjuvant liposome delivery system enhanced generation of antigen-specific Th1 cells and CD8+ T cells in newborn mice.

#### Signaling Pathways and *In Vitro* Modeling

Recent advances in the field of innate immunity have led to our increased understanding of mechanisms involved in vaccine-induced immunity, which has informed rational vaccine design (64, 65). Accordingly, dissecting innate immune signaling pathways along with improving *in vitro* models are crucial for characterization and evaluation of novel antigen and adjuvants.

Dr. Ivan Zanoni (Boston Children’s Hospital; Harvard Medical School) presented work on how the physical properties of PAMPs govern the immune response. The activation of PRRs in innate immune cells by PAMPs or DAMPs is regarded as a crucial step for induction of adaptive immunity. Migratory dendritic cells (DCs) play an essential role in initiating antigen-dependent adaptive immune response. Dendritic cells dispersed throughout peripheral tissues sense the presence of microbial clues released during an infection, are activated, and migrate to the draining lymph node (dLN)—enabling a transfer of “information” from peripheral tissue to the dLN—where an antigen-dependent adaptive immune response against the pathogen is initiated. Activation of PRRs allows migration of DCs from the periphery into the draining lymph node (dLN) where they transfer the information from the inflamed/infected tissue into the dLN and thus trigger activation of T-cell and B-cell responses. In last few years, beside this antigen-dependent response in dLN, another event antigen-independent innate response in LN has been discovered. Several groups have shown that inflammation in the periphery enables migration of DCs that interact with stromal cells which allows for lymph node expansion to create physical space for T- and B-cells to proliferate and establishment of a pro-inflammatory milieu, sustain and allow a potent adaptive innate response. Dr. Zanoni’s laboratory made the observation that although soluble fungal polysaccharides remain immune-silent in the periphery, they become potent immunogens in the dLN. These fungal moieties activate an immune response similar to viral infection, without any requirement for phagocyte migration. These observations highlight how the physical form of certain PAMPs impacts innate and adaptive immunity (66).

Dr. Leif Erik Sander (Charité University Hospital, Berlin, Germany) highlighted how recognition of microbial viability serves as a key driver of vaccine responses. Previous work by Dr. Sander established how murine antigen-presenting cells can discriminate living from dead bacteria (67, 68). In particular, microbial RNA can mimic microbial infections therefore highlighting the need to target nucleic acid sensing receptors to promote robust T-cell dependent immunity (69). Because these mRNAs are not found in dead bacteria, they belong to a special class of PAMPs, which have been termed “vita-PAMPs”. Accordingly, live microbes activate multifaceted immune responses that facilitate long-lasting protective immunity. Future vaccine development pipelines may integrate vita-PAMP activation strategies to enhance immunization, especially for infectious diseases for which no current vaccine exists.

### Vaccines for Non-infectious Indications

Dr. Joost Oppenheim (Center for Cancer Research, National Cancer Institute, NIH, USA) presented work on therapeutic vaccination for tumors in mice. Over the last 15 years, research from Dr. Oppenheim’s lab and other groups have identified numerous cytokine-like products of cell which are constitutively present and, apart from functioning as danger signals, are involved in host defense, inflammation as well as repair mechanism, called pathogen‐associated molecular pattern molecules (PAMPs) and damage‐associated molecular pattern molecules (DAMPs). Both innate and adaptive immune responses are engaged by the alarmins’ (PAMPs and DAMPs) activation of the dendritic cells (70). In particular, the alarmin HMGN1 (High Mobility Group Nucleosome Binding Domain 1) which intracellularly acts as non-histone chromosome protein that regulates chromatin remodeling and transcription of certain genes, on the contrary, extracellularly acts as antimicrobial and Th1-polarizing signal. HMGN1, which acts on TLR4 to induce DC migration and maturation, is therefore crucial for host defense mechanisms. Multiple studies from Dr. Oppenheim’s group have shown how HMGN1 contributes to the generation of antitumor immunity and has therefore drawn interest as a potential target for antitumor therapy (71). They made the observation that synergistic effect of R848 (TLR7/8 agonist) along with HMGN1 (TLR4 agonist) leads to augmented IL-12 responses and phenotypic maturation of mouse bone marrow derived dendritic cells (BMDCs) (72). This led to the development of a therapeutic vaccine (TheraVac) combining HMGN1 and R848 plus a checkpoint inhibitor (Cytoxan), which is effective on large tumors in mice without use of exogenous tumor-associated antigen(s) (73).

Dr. Thomas Kosten (University of Houston, Texas) summarized his more than two decades of work on anti-addiction vaccines (74, 75). According to a 2018 substance use survey, ~165 million people aged ≥12 years in the USA (~60% of the population) were past month substance users (*e.g.*, tobacco, alcohol, or illicit drugs such as opioids). Substance use disorder (SUD) is a growing problem with severe outcomes such as overdose-related death, yet there are limited options presently available to overcome this issue. Anti-addiction vaccines are aimed at inducing antibodies against a given drug (*e.g.*, opioid) and trap it in the blood stream. These antibody–drug complexes are unable to enter the blood–brain barrier and are subsequently removed from the circulation, thereby making drugs non-reinforcing and also preventing overdose-related death due to respiratory suppression. Although there are no currently approved anti-addiction vaccines, limited safety concerns and advances in efficacy make anti-addiction vaccines a promising therapeutic strategy to counteract the increasing clinical burden of SUDs.

## Funding Perspectives

A panel discussion regarding funding perspectives moderated by Dr. Ofer Levy included representatives from institutions that support vaccine research. Dr. Mary Marovich (Director of Vaccine Research Program; Division of AIDS, NIH) discussed NIH’s current perspective on HIV vaccine development. This included NIH’s current interest in potential vaccines which induce broadly neutralizing antibodies to target HIV, funded by the Pre-clinical Research and Development Branch (PRDB) and Vaccine Translational Research Branch (VTRB) portfolios.

Dr. Mercy PrabhuDas (Program Officer in the Division of Allergy, Immunology and Transplantation at NIAID, NIH) discussed the interest at NIAID in vaccine research tailored for vulnerable populations, including the Immunity in Neonates and Infants and the Immunity in the Elderly programs at NIAID.

Dr. David Kaufman (Chief Medical Officer at the Bill & Melinda Gates Medical Research Institute) discussed innovative approaches to accelerate discovery and development of vaccines for global populations in need, including utilizing systems immunology tools, molecular epidemiology studies, controlled human infection models, and adaptive trial designs to optimize vaccine dosing, as well as creation of more stringent and accurate go/no-go decision points.

## Towards Precision Vaccinology

**Figure 2:**
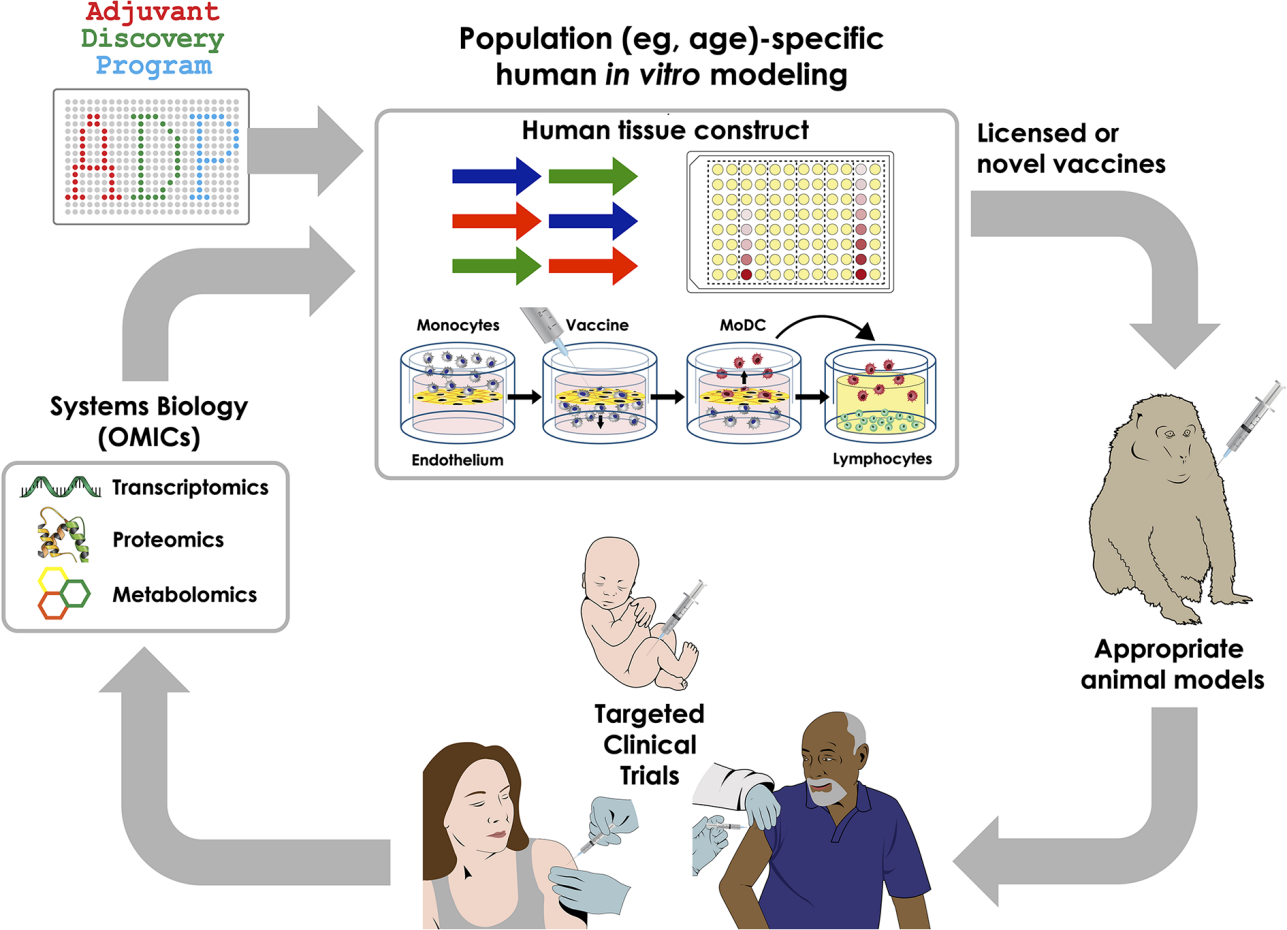
Integrated approaches to precision vaccinology. Discovery and development of precision vaccines may include a series of sequential approaches including system biology analysis of biosamples from clinical trials to define vaccine-induced cellular and molecular signatures that correlate with immunogenicity, thereby generating new mechanistic hypotheses; use of human *in vitro* systems for hypothesis testing and targeted adjuvant discovery employing population-specific biosamples, selection of appropriate animal models and clinical trials in specific vulnerable populations.

Vaccinology has largely been driven by an empiric paradigm that assumes that a given vaccine will work equally in all individuals. Accordingly, until recently, few vaccines had been specifically tailored to overcome the immunosenescence of aging or altered efficacy in immunocompromised individuals (76). Recent innovations in genetic engineering, human *in vitro* modeling, formulation science, and systems biology have fueled rapid advances in vaccine research that accounts for demographic factors (*e.g.*, age, sex, genetics, and epigenetics) in vaccine discovery and development. These novel approaches are being leveraged to discover and develop vaccinal antigens and, as needed, adjuvant systems to enhance vaccine immunogenicity while maintaining safety. The potential benefits of these research efforts can only be fully utilized if they also help promote vaccine confidence and are coupled with universal vaccine coverage (77). These approaches are ushering in an era of precision vaccinology aimed at tailoring immunization for vulnerable populations with distinct immunity (**Figure 2**). In 2004 Dr. Stanley Plotkin, referred to as the ‘Godfather of Vaccinology’, outlined the history of the first five revolutions in vaccinology and outlined some predictions for the next candidate “sixth revolution” (9). In the past two decades, advancements in novel *delivery systems* have led to remarkable improvements in vaccine translation, positioning it as a sixth revolution in vaccinology (26). During the conference’s conclusion panel, he emphasized how the concept of precision vaccines represents the “seventh revolution” in vaccinology wherein the attention of vaccine development is focused on the individual (or group) rather than the herd. Specifically, instead of focusing on development of vaccines for use in large populations to control epidemic diseases the focus has now shifted to the individual characteristics of each vaccinee, including assessment of efficacy and safety of the vaccine on an individual basis. Indeed, some of the currently administered vaccines are not fully protective in specific populations (*e.g.*, influenza and mumps vaccine) (27, 78). An individual’s characteristics such as prior exposure, priming, immune status (for live vaccines) are critical for efficacy of influenza vaccines. Additionally, multiple recent mumps outbreaks in colleges and closed-knit communities, including among vaccinated individuals (79), and lack of appropriate correlates of protection (80) have highlighted the need to develop vaccines in an individualistic manner. In regard to safety, rare severe reactions, such as Guillan–Barré Syndrome after exposure to influenza vaccine or virus and intussusception after rotavirus vaccination, still need to be addressed (78). Therefore, under rare circumstances, certain vaccines (*e.g.*, live vaccines) may be contraindicated in certain individuals, depending upon their immune disposition. He also highlighted the need to individualize adjuvants, referring to a recent example where an adjuvant system-03 (AS03; an oil-in-water emulsion adjuvant)-adjuvanted influenza vaccine was associated with increased risk for narcolepsy only for vaccinated populations in Scandinavia, suggesting population-specific vaccine effects (78). The Center for Disease Control (CDC) recently assessed safety data on adjuvanted pH1N1 vaccines (*arepanrix*-AS03, *Focetria*-MF59, and *Pandemrix*-AS03) from 10 global study sites and confirmed, other than the association for *arepanrix* in Scandinavia, lack of any detectable associations between the vaccines and narcolepsy (81). However, the phenomena observed in a particular population (Scandinavian study subjects) further emphasized the need for evaluation of adjuvanted vaccines in a population-specific manner to enhance safety and efficacy.

In conclusion, the second biennial IPVC 2019 reviewed and incorporated contemporary topics guiding the field of precision vaccines. As with any conference, there were areas that were important but not covered which may be potential topics for discussion at the next IPVC (2021), including: A) measuring vaccine efficacy: despite our increased understanding of immune responses to infection, there is incomplete understanding of the immune responses specifically required for protection; B) implementation research that focuses on technical, managerial, financial, systems, socio-behavioral, and communication aspects to inform evidence-based policies and practices important for introduction of new vaccines and maintaining/expanding coverage for currently approved vaccines; C) vaccine access: wider global availability of affordable vaccines is required to curb preventable diseases; and D) vaccines for allergy: there is growing interest in utilizing vaccines for allergen-specific immunotherapy and for disease-modifying allergy therapy (82). The third International Precision Vaccines Conference, to be held on September 22^nd^ and 23^rd^ of 2021 at Harvard Medical School (Boston, MA, USA), will consider these and other areas by gathering a multidisciplinary community of scientists to review progress and encourage partnerships focused on the most current challenges and opportunities in precision vaccines research.

## Author Contributions

DS, SVH, OTI, JTE, JD-A, DD, and OL wrote the manuscript. OL edited the manuscript. All authors contributed to the article and approved the submitted version.

## Funding

The PVP is supported in part by US National Institutes of Health (NIH)/National Institutes of Allergy and Infectious Diseases (NIAID) awards including Molecular Mechanisms of Combination Adjuvants (1U01AI124284-01), Adjuvant Discovery (HHSN272201400052C and 75N93019C00044) and Development (HHSN272201800047C) Program Contracts, and Human Immunology Project Consortium (HIPC) U19AI118608-01A1 to OL. DD’s laboratory is supported by NIH grant 1R21AI137932-01A1 and Adjuvant Discovery Program contract 75N93019C00044 as well as by the BCH Department of Pediatrics and the Chief Scientific Office.

## Conflict of Interest

DD, OL, and JTE are named inventors on several patent applications related to vaccine adjuvants. JTE is currently employed by Inimmune corporation. OTI is currently employed by Sanofi Pasteur but was not at the time of gathering this data.

The remaining authors declare that the research was conducted in the absence of any commercial or financial relationships that could be construed as a potential conflict of interest.
